# Bipolar radiofrequency catheter ablation for refractory perimitral flutter: a case report

**DOI:** 10.1186/s12872-015-0132-z

**Published:** 2015-10-28

**Authors:** Kenichiro Yamagata, Dan Wichterle, Petr Peichl, Bashar Aldhoon, Robert Čihák, Josef Kautzner

**Affiliations:** Department of Cardiology, Institute for Clinical and Experimental Medicine (IKEM), Vídeňská 1958/9, Prague, 140 21 Czech Republic

**Keywords:** Catheter ablation, Radiofrequency, Bipolar ablation, Perimitral flutter

## Abstract

**Background:**

Mitral isthmus is often targeted as a part of stepwise approach during radiofrequency ablation for persistent atrial ablation. Acute success rate in achieving the mitral isthmus block is only modest, late reconduction rate is relatively high and, consequently, incomplete lesion may be proarrhythmic. We describe the first-in-man experience with successful MI ablation by bipolar RF energy delivery.

**Case presentation:**

A 64-year-old caucasian man after two previous ablation procedures for drug resistant atrial fibrillation in recent four years, which included pulmonary vein isolation and linear left atrial lesions, was referred for the treatment of recurrent perimitral flutter. Despite the third attempt to create bidirectional block at the mitral isthmus region, we were not even able to stop the arrhythmia by aggressive unipolar radiofrequency ablation both from the left atrium and coronary sinus, because of deeply embedded slow conducting channel probably around the vein of Marshall. Arrhythmia was finally terminated and the block was achieved by bipolar radiofrequency ablation between two irrigated-tip catheters positioned at the left atrial endocardium and contralaterally inside the coronary sinus.

**Conclusion:**

Bipolar radiofrequency energy delivery can be an option for ablation of perimitral flutter resistant to standard unipolar radiofrequency ablation. This may improve clinical outcome of patients undergoing non-pharmacological treatment for persistent atrial fibrillation. The safety and efficacy of this technique has to be confirmed in future studies.

## Background

Radiofrequency (RF) catheter ablation for persistent atrial fibrillation is often a challenging procedure and a stepwise approach that incorporates linear lesions is frequently used to terminate the arrhythmia [1]. Mitral isthmus (MI) between the left inferior pulmonary vein and the mitral annulus is often targeted. However, acute success rate in achieving the MI block is only modest, late reconduction rate is relatively high and, consequently, incomplete lesion may be proarrhythmic [1].

In animal experiments, bipolar RF ablation was proposed to increase the lesion depth and narrow the lesion width compared to conventional unipolar RF setting [2–5]. Its clinical efficacy has already been reported for elimination of accessory pathways, septum-related atrial tachycardias, intramural scar-related ventricular tachycardias, and refractory outflow tract ventricular arrhythmias [5–9].

We describe here a case of MI-dependent atrial tachycardia that recurred after two previous ablation procedures for persistent atrial fibrillation targeting the MI region. Despite the third attempt to create bidirectional block at MI we were not even able to stop the arrhythmia by conventional unipolar ablation. Arrhythmia was finally terminated and the block was achieved by bipolar RF ablation between two irrigated-tip catheters positioned at the left atrial (LA) endocardium and inside the coronary sinus (CS).

## Case presentation

A 64-year-old man with a history of hypertension and structurally normal heart was referred to our center for the treatment of persistent atrial fibrillation refractory to Class Ic antiarrhythmic drug. RF ablation procedure was performed with a 3.5 mm irrigated tip catheter (Navistar Thermocool, Biosense Webster, Diamond Bar, CA, USA) via nonsteerable transseptal sheath, navigated by electroanatomic imaging (CARTO 3, Biosense Webster) and facilitated by intracardiac echocardiography (AcuNav, Siemens Healthcare, Erlangen, Germany). Pulmonary veins were isolated and extensive LA ablation was performed including roof line, MI line and ablation of complex fragmented atrial electrograms in LA and CS. Atrial fibrillation converted to a clockwise perimitral flutter. However, we were not able to terminate flutter by further ablation and also the attempt to achieve electrical block at the MI after DC cardioversion failed when using our standard RF setting of 30–35 W with the irrigation flow of 20 ml/min (Cool Flow pump, Biosense Webster) at LA endocardium and 20–25 W with the irrigation flow of 30 ml/min within the CS with a temperature limit of <40 °C (EP Shuttle, Stockert, Freiburg, Germany).

Three years after the index procedure, the patient started to suffer from the recurrence of persistent atrial tachycardia. At the second ablation procedure, durable isolation of all pulmonary veins was confirmed and the mechanism of the atrial tachycardia was established as a double-loop reentry with a counter-clockwise circuit around the mitral annulus and clock-wise circuit around the left pulmonary veins. After a significant effort we succeeded to finalize a bidirectional block at both LA roof and MI lines.

**Fig. 1:**
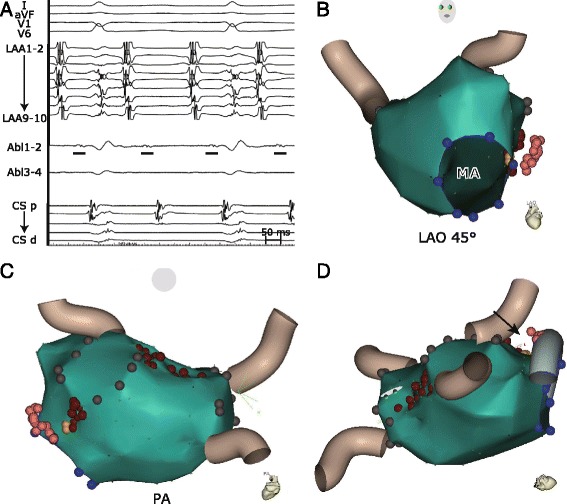
Perimitral flutter - electrograms and 3D imaging. **a**: Fractionated electrograms (marked by underline) were recorded by the ablation catheter (Abl) positioned at the MI. They belong to slow-conducting zone of clockwise perimitral flutter. **b**-**d** Electroanatomic shell of the LA after roof and MI line ablation. Left anterior oblique (LAO, 45°) (**b**) and postero-anterior (PA) view (**c**) with the left inferior pulmonary vein hidden to visualize the MI. Left superior oblique view (**d**) shows the free space area (*black arrow*) between LA endocardium and CS. CARTO tags definition: yellow = successful bipolar ablation point at the endocardium, dark red = unipolar ablation point at the endocardium, pink = unipolar ablation point within the CS, blue = mitral annulus, gray = scar. LAA = left atrial appendage

Despite this result, the patient experienced a recurrence of persistent atrial tachycardia one year later (Fig. 1a). The LA roof was reablated and the conduction block was easily restored. Based on electroanatomic activation and entrainment mapping, a residual atrial tachycardia was clockwise perimitral flutter. Ablation of posterior portion of the MI from both the endocardium (with very low-voltage fragmented far-field electrograms) and within the CS (with virtual absence of local signals) with cumulative RF time exceeding 800 s only slightly prolonged the cycle length of tachycardia (Fig. 1a, b, c). There was a considerably large non-accessible space between LA endocardium and CS according to electroanatomic map which indicated a significant bulk of interposed tissue (with estimated endo-epicardial distance of minimum 11 mm, Fig. 1d). This anatomy prevented elimination of deeply embedded slow conducting channel, probably around the vein of Marshall. The distance of 11 mm might have been even underestimated due to anatomy distorsion because of contralateral tissue compression/dislodgement by the mapping catheter. At this stage of the procedure, we decided to convert to bipolar RF ablation.

**Fig. 2:**
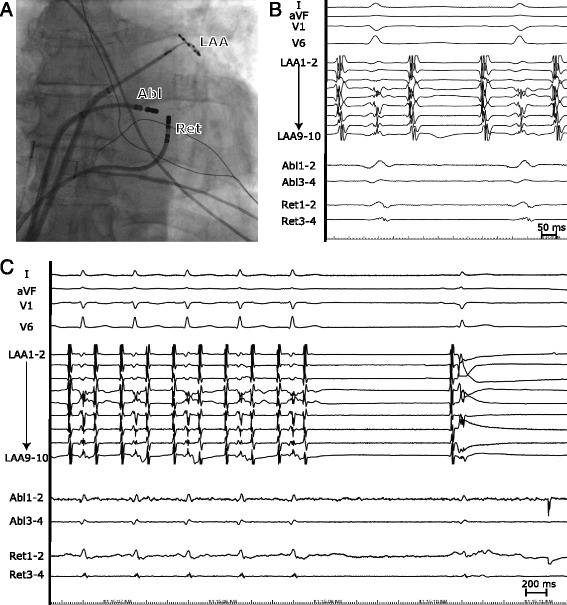
Bipolar ablation of mitral isthmus. **a** Anterior–posterior fluoroscopic image of the catheter position. Ablation (Abl), return electrode (Ret) and lasso catheter is positioned at the LA endocardium, within the CS and at the left atrial appendage (LAA) respectively. **b** Electrograms at the successful bipolar ablation site. **c** Termination of the flutter by bipolar ablation

We used a second 3.5 mm irrigated tip ablation catheter (Celsius Thermocool, Biosense Webster) which replaced the diagnostic decapolar CS catheter. It was connected to a purpose-made switchbox in order to display the local electrograms and to connect catheter tip as a return electrode to the RF generator instead of dispersive patch. The catheter was visualized in real time by impedance-based method of CARTO 3 system, navigated via CS to the MI level and deflected toward the LA wall. The Navistar Thermocool was positioned endocardially to the posterior portion of the MI where low-voltage fragmented far-field electrograms were tagged prior to unipolar ablation (Fig. 2a). Of note, no local signals were visible at both ablation sites due to prior extensive ablation at the MI and development of local edema (Fig. 2b). We set the RF power to 30 W with the single-pump irrigation flow of 60 ml/min which was equally distributed to both ablation catheters with an assumption of identical resistance of the irrigation systems.

After 25 s of RF energy delivery (with an impedance drop of 30 ohms), the tachycardia terminated and sinus rhythm was restored (Fig. 2c). At the same time, bidirectional conduction block was achieved as assessed by differential pacing criteria. Safety bipolar burns (cumulative RF time of 100 s) were applied to the close vicinity of the success site. After waiting time of 20 min, conduction block persisted and other arrhythmias were not inducible by atrial burst pacing up to the cycle length of 200 ms. Patient did not experience the recurrence of atrial tachycardia during the follow up of 10 months.

## Discussion

This case report illustrates difficulties that may occur when ablating the MI for perimitral flutter and describes for the first time the use of bipolar RF ablation in this region.

## Mitral isthmus block

Anatomical obstacles to successful ablation of the MI include variable myocardial thickness across the isthmus (sometimes with pouch or cleft), isthmus length, convective cooling by local blood vessels, myocardial sleeves around the CS, and epicardial connections along the vein/ligament of Marshall. In prior randomized control studies, acute success rates in MI ablation were reported between 31 % and 88 % with frequent need for CS ablation [1].

In order to facilitate the MI block, CS occlusion by inflating an air-filled balloon to reduce the cooling effect of CS blood flow has been proposed. In a single-center randomized study, this technique reduced the need for CS ablation and total RF time, though the acute success rate was not significantly different [10]. The impact of steerable sheaths, which improve catheter stability and navigation at the MI, was investigated in a single-center randomized study. Their use significantly improved the efficacy of MI ablation with success rate of 98 % vs 78 % with the use of conventional sheaths [11]. Recently, ethanol ablation of the vein of Marshall in a combination with unipolar RF ablation showed a success rate of 100 % in achieving the MI block if the vein of Marshall could be cannulated [12]. However, this is a demanding technique which was not attempted in our case.

Anterior ablation line (connecting anteroseptal mitral annulus with isolated left/right PV ostia or roof-block line) is another strategy to achieve the perimitral block. This is sometimes useful in patients with pre-existing severe anterior LA scarring when additional ablation can be expected for localized AF/AT sources at anterior LA on top of ablation for perimitral reentry. We use this infrequently because true block at that line significantly delays LAA activation in sinus rhythm which in turn may interfere with the LAA mechanical function, specifically with timely contribution to LV filling. In our patient, who had relatively healthy LA at baseline, empirical lateral mitral isthmus ablation was performed as part of stepwise strategy for persistent AF already during the index procedure. In such a situation, switching to anterior line during the re-do procedures may be even more harmful provided that there is already slow conducting zone at mitral isthmus which may result in even more delayed LAA emptying after achievement of the anterior block.

## Bipolar ablation

Transmurality of unipolar RF lesions is limited by the local tissue thickness. Bipolar RF ablation with the use of two catheters placed from opposite aspects of targeted region has been reported to make consistently deeper or continuous lesions [2–5].

Anecdotal reports on clinical utility of bipolar ablation come already from the era of non-irrigated catheters. Left posterolateral accessory pathway was modified by bipolar RF ablation between CS and ventricular aspect of mitral annulus [6]. Bashir et al. reported a series of 8 cases of successful bipolar RF ablation of resistant posteroseptal accessory pathways with two catheters positioned opposite to each other at the mitral and/or tricuspid annulus [7]. Idiopathic ventricular tachycardia was eliminated by bipolar RF ablation between the left aortic sinus and the subvalvular site in the left ventricle [8].

Recently, irrigated-tip bipolar ablation (after failed unipolar ablation) was used with reasonable acute success rates for the treatment of septum-related atrial arrhythmias [5], intramural ventricular tachycardias in structural heart disease with both paraseptal and epi-endocardial configuration of ablation catheters [5], and refractory outflow tract ventricular ectopy [9].

## Bipolar ablation at the mitral isthmus region

There is only one experimental study on bipolar RF ablation along the mitral annulus which was performed in excised superfused swine hearts [13]. Bipolar ablation was performed between irrigated-tip ablation catheter (25 W, 120 s, 20 ml/min) at multiple endocardial sites and septapolar diagnostic CS catheter with 4-mm nonirrigated electrodes, and was compared with unipolar endocardial ablation. Complete transmural lesion along the ablated region according to macroscopic examination was achieved in 75 % and 0 % cases by bipolar and unipolar RF ablation, respectively, with corresponding rate of steam pop of 37.5 % and 0 %.

There are differences between this animal study and the presented clinical case. First, we targeted the remaining conduction channel hidden deeply in the tissue anteroinferior to the left inferior pulmonary vein while mitral annulus region was ablated in the animal study. Consequently, a vector of our bipolar ablation was less perpendicular to the LA wall. Second, we delivered bipolar RF energy into the thick postablation fibrotic tissue, which is empirically less prone to the steam pop compared to healthy perimitral tissue in the animal study. Third, we ablated region between two irrigated catheter tips while in the animal study, irrigated tip catheter was used at the LA endocardial site only and all electrodes of septapolar CS catheter were interconnected to create a single return electrode array. Fourth, the duration of our bipolar ablations was, for safety reasons, limited to <40 s compared to 120 s in the animal study. Fifth, cooling effect of CS blood flow was missing in the animal experiment.

Optimum setting for bipolar RF ablation of the MI is obviously unknown. Because of limited pilot data, we set RF power similar to that previously used for ablation of atypical flutters originating at interatrial septum [5] with high-flow irrigation from both sides (not reported before). Tip of ablation catheter inside the CS was bent towards the atrial wall in order to avoid the RF energy delivery in the direction of the CS free wall and, thus, minimize the risk of perforation in case of inadvertent steam pop. We also observed the targeted region by intracardiac echocardiography for early detection of tissue overheating with the risk of imminent steam pop. The arrhythmia terminated rather late but we cannot exclude slight change in catheter positions during the ablation. Generally, we believe that bipolar RF ablation has a potential to create better focused (narrower) lesion compared to unipolar ablation. This may help avoiding extensive ablation of otherwise healthy myocardium for difficult-to-achieve ablation endpoint, as well as reducing the collateral organ damage. In this respect, injury of left circumflex coronary artery (LCx) is probably the main issue. While asymptomatic, thermally induced spasms of LCx were noticed quite frequently after unipolar MI ablation [14], LCx occlusion requiring intervention was rather rare event (0.2–0.5 %) in three large cohorts [15–17] especially with high-power setting and the use of steerable sheaths. It is plausible to speculate that LCx injury might be more frequent with the use of bipolar RF ablation and our single uneventful case does not tell anything about safety of this technique. The risk for esophageal injury, which can also be associated with CS ablation, may theoretically be reduced with bipolar compared unipolar ablation because maximum tissue heating concentrates in the atrial wall between both catheter tips. For future bipolar MI ablation, posterolateral MI should be targeted where the risk and consequences of LCx injury are perhaps lower compared to anterolateral MI [1], and the maximum power of 30 W could be advocated with careful vigilance for acute complications.

## Limitations

First, the follow up period of 10 months is too short (with respect to previous delayed arrhythmia recurrences) to suggest the durability of MI block. Second, we did not use steerable sheath and contact force sensing ablation catheter, which might facilitate reaching the ablation endpoint even with unipolar RF energy. In this respect, perhaps steerable sheaths alone are of greater importance because the knowledge of incidental inadequate contact force does not guarantee that desirable and stable contact at that site can be achieved.

## Conclusion

To the best of our knowledge, this is the first-in-man experience with the MI ablation by bipolar RF energy, which suggests that such approach can be an option for ablation of perimitral flutter resistant to unipolar RF energy delivery. Conversion from standard to bipolar ablation can easily be performed at any stage of ablation procedure. The safety and efficacy of this technique has to be confirmed in future studies.

## Consent

Written informed consent was obtained from the patient for publication of this Case report and any accompanying images. A copy of the written consent is available for review by the Editor of this journal.

## Abbreviations

CS: Coronary sinus
LA: Left atrium
LAA: Left atrial appendage
LAO: Left anterior oblique
LCx: Left circumflex coronary artery
MI: Mitral isthmus
PA: Posteroanterior
RF: Radiofrequency
